# Computer-Aided Tomographic Analysis of Interstitial Lung Disease (ILD) in Patients with Systemic Sclerosis (SSc). Correlation with Pulmonary Physiologic Tests and Patient-Centred Measures of Perceived Dyspnea and Functional Disability

**DOI:** 10.1371/journal.pone.0149240

**Published:** 2016-03-01

**Authors:** Fausto Salaffi, Marina Carotti, Eleonora Di Donato, Marco Di Carlo, Luca Ceccarelli, Gianmarco Giuseppetti

**Affiliations:** 1 Department of Reumatology, Polythecnic University of Marche, Jesi (Ancona), Italy; 2 Department of Radiology, Polythecnic University of Marche, Ancona, Italy; Nippon Medical School Graduate School of Medicine, JAPAN

## Abstract

**Objectives:**

This study was designed (a) to evaluate an improved quantitative lung fibrosis score based on a computer-aided diagnosis (CaM) system in patients with systemic sclerosis (SSc),—related interstitial lung disease (SSc-ILD), (b) to investigate the relationship between physiologic parameters (forced vital capacity [FVC] and single-breath diffusing capacity for carbon monoxide [DLCO]), patient-centred measures of dyspnea and functional disability and CaM and visual reader-based (CoVR) methods, and (c) to identify potential surrogate measures from quantitative and visual HRCT measurement.

**Methods:**

126 patients with SSc underwent chest radiography, HRCT and PFTs. The following patient-centred measures were obtained: modified Borg Dyspnea Index (Borg score), VAS for breathing, and Health Assessment Questionnaire-Disability Index (HAQ-DI). HRCT abnormalities were scored according to the conventional visual reader-based score (CoVR) and by a CaM. The relationships among the HRCT scores, physiologic parameters (FVC and DLCO, % predicted) results and patient-centred measures, were calculated using linear regression analysis and Pearson’s correlation. Multivariate regression models were performed to identify the predictor variables on severity of pulmonary fibrosis.

**Results:**

Subjects with limited cutaneous SSc had lower HAQ-DI scores than subjects with diffuse cutaneous SSc (p <0.001). CaM and CoVR scores were similar in the 2 groups. In univariate analysis, a strong correlation between CaM and CoVR was observed (p <0.0001). In multivariate analysis the CaM and CoVR scores were predicted by DLco, FVC, Borg score and HAQ-DI. Age, sex, disease duration, anti-topoisomerase antibodies and mRSS were not significantly associated with severity of pulmonary fibrosis on CaM- and CoVR methods.

**Conclusions:**

Although a close correlation between CaM score results and CoVR total score was found, CaM analysis showed a more significant correlation with DLco (more so than the FVC), patient-centred measures of perceived dyspnea and functional disability. Computer-aided tomographic analysis is computationally efficient, and in combination with physiologic and patient-centred measures, it could allow a means for accurately assessing and monitoring the disease progression or response to therapy.

## Introduction

Systemic sclerosis (SSc) is a heterogeneous complex of diseases characterized by multiorgan involvement, endothelial dysfunction, excessive collagen production and immune system abnormalities [1]. Clinically, patients can have diverse systemic manifestations with any combination of skin, pulmonary, cardiac, renal, musculoskeletal and gastrointestinal involvement. Interstitial lung disease (ILD) is a devastating and significant cause of death in patients with SSc. In early autopsy studies, up to 100% of patients were found to have parenchymal involvement [1]. Parenchymal lung involvement often appears early after the diagnosis of SSc, with 25% of patients developing clinically significant lung disease within 3 years as defined by physiological, radiographic or bronchoalveolar lavage abnormalities [2]. Progressive decrements in lung function of patients with symptomatic lung disease are also accompanied by a decline in their emotional well-being and in their ability to perform day-to-day activities, that is, their health-related quality of life (HRQoL) [3]. Baseline diffusing capacity for carbon monoxide (DLco) and forced vital capacity (FVC) levels have traditionally been used as measures of disease severity and reductions in both parameters have been associated with increased mortality in the SSc-ILD.

High resolution computed tomography (HRCT) has now become an important part of the routine detection and evaluation of SSc-ILD [4,5]. It has been shown to be more accurate than chest radiography in detecting and characterizing diffuse lung diseases, and abnormalities on HRCT correlate more closely with physiologic parameters (FVC and DLco) [6, 7]. HRCT features of fibrosis are present in 55% to 65% of all patients with SSc and in up to 96% of those with abnormal pulmonary function tests (PFTs) results [8,9]. Although formal CT scoring is not realistic in routine practice, rapid semi-quantitative estimation of disease extent on CT in combination with a FVC threshold has been used to stage disease as limited or extensive [10–13]. To date, several computer tools to automatically segment the lung, using HRCT images, have been developed. They include image display (e.g., multiplanar reformations and surface shading for three-dimensional and volume rendering), anatomic image quantitation (e.g., area and volume of airways and lungs) and regional characterization of lung tissue (analysing attenuation, changes in attenuation, and texture patterns in the imaged lung) [13, 14]. With respect to the traditional visual interpretation of HRCT lung findings, the automatic computer-based assessment may improve the objectivity, sensitivity, and repeatability of quantitative changes in the lung features [14–16]. Previously we showed a high agreement concerning the semi-quantitative HRCT analysis performed by experienced radiologists, and a significant association between the descriptive parameters by both the quantitative OsiriX assessment and the HRCT semi-quantitative analysis [17]. More recently, we investigated the performance of a computer-aided method (CaM) for the quantification of ILD, in seventy-nine patients with SSc, in terms of correlation regarding both the conventional visual reader-based score (CoVR) and the physiologic parameters, feasibility and inter-reader reliability of the CaM [18]. The results indicate that the CaM analysed by OsiriX provides a good concurrent validity, reliability and feasibility for the assessment of SSc-ILD.

The three purposes of our study were: (a) to evaluate an improved quantitative lung fibrosis score based on CaM system in patients with SSc-ILD, (b) to investigate the relationship between physiologic parameters (FVC and DLco), patient-centred measures of dyspnea and functional disability and CaM and CoVR methods, and (c) to identify potential surrogate measures from quantitative and visual HRCT measurement.

## Patients and Methods

### Study population

This cross-sectional study was approved by our institutional review board of the Zona Territoriale 5 ASUR Marche. The participants provided a verbal consent to participate in this study. A written consent was not obtained because HRCT, PFTs and the other tests applied to participants are the current clinical practice used in our Departement for the evaluation of patients with SSc. We recorded the consent on the clinical record of the patients and the ethics committee approved this procedure. Patients with SSc, defined by the American College of Rheumatology (formerly, the American Rheumatism Association) classification criteria [19], were included in the study. SSc patients were classified in limited and diffuse cutaneous involvement (lcSSc and dcSSc, respectively). LcSSc was characterized by thickening of the skin distal to the elbows and knees and proximal to the clavicles (including the face) whereas dcSSc was characterized by thickening of the skin proximal as well as distal to the elbows and knees and including the trunk and the face subtype [20]. The modified Rodnan skin score (mRSS) was used for the assessment of skin damage in patients with SSc [21]. Scores of 0 (no thickening), 1 (mild thickening), 2 (moderate thickening), and 3 (severe thickening) are given to each area and added up to a total score from 0 (best) to 51 (worst). The presence of autoantibodies, including anti-topoisomerase I and anti-centromere was also investigated. Exclusion criteria included: absence of recent or current respiratory infection, severe pulmonary hypertension requiring specific treatment with either bosentan or epoprostenol, uncontrolled congestive heart failure or clinically significant abnormalities other than ILD identified on chest radiography or on HRCT. Echocardiogram and right heart catheterization are not included as a routine part of the visit. Only a small group of subjects (24.6%) had undergone echocardiography at enrollment, so the results of these studies were not included in our analysis.

### Patient-centred measures

The following patient-centred measures were obtained: dyspnea assessment using the modified Borg Dyspnea Index (Borg score) by using the interviewer-administered paper version [22], the Health Assessment Questionnaire-Disability Index (HAQ-DI) [23, 24], which is a generic, self-administrated patient-reported outcome (PRO) instrument targeted for musculoskeletal impairment, as well as the Visual Analogue Scale for breathing (VAS for breating) problems interfering with physical activities (range 0–10). Borg score is a numerical scale for assessing perceived dyspnea (breathing discomfort) with a scale of 0 = no breathlessness at all, 0.5 = very very slight (just noticeable), 1 = very slight, 2 = slight breathlessness, 3 = moderate, 4 = somewhat severe, 5 = severe breathlessness, 7 = very severe breathlessness, 9 = very, very severe (almost maximum) and 10 = maximum [22]. The VAS for breathing allows patients to self-assess their degree of difficulty in performing daily activities due to shortness of breath. A continuous 100-mm scale (from no limitation of activity to severe limitation of activity) is used for this assessment [25]. The HAQ-DI is a condition-specific measure of functional status (assessing activities of daily living), intended for use in arthritis [23]. The standard HAQ-DI is calculated as an ordinal variable, from 0 = no disability to 3 = severe disability. It has been found to correlate with cutaneous and visceral involvement in SSc at baseline and with changes in physiologic parameters over time [26, 27].

### Pulmonary function tests

PFTs were performed within 2 weeks since the execution of the HRCT scan by a flow-sensing spirometer and a body plethysmograph connected to a computer for data analysis. PFTs were performed while the patient was at rest in a seated position. These tests consisted in spirometry using a computerised lung analyser (MasterScreen Diffusion, Jaeger GmbH, Höchber, Germany).

FVC (% predicted value) and DLCO (% predicted value, corrected for haemoglobin) were obtained. The PFTs performed were based on published guidelines [28]. At least three measurements were taken for each variable to guarantee repeatability.

### HRCT assessment and visual reader-based disease quantification

All patients underwent volumetric thin-section CT examinations using a CT 64 GE light Speed VCT power scanner. Scans were obtained at full inspiration from the apex to the lung base with the patients in the supine position. Scanning parameters were: 120 kV, and 300 mAs, acquisition time 0.8 s, slice thickness 1 mm with 0.6 mm reconstructions and the smallest possible field of view (FOV) covering both lungs. The scans were viewed with a window level of -600 Hounsfield units (HU) and width of 1600 HU. HRCT assessment did not include the use of contrast media agents. The parenchymal abnormalities on HRCT were coded and scored by two independent readers, blinded to the results, according to Warrick et al [11]. A point value was assigned to each abnormality as follows: ground-glass appearance = 1, irregular pleural margins = 2, septal/subpleural lines = 3, honeycombing = 4, subpleural cysts = 5. In each patient the "severity of disease" score was obtained by adding single point values. The mean values of the two independent readers were used as a final control group. An "extent of disease" score was obtained by counting the number of bronchopulmonary segments involved for each abnormality: one to three segments scored as 1; four to nine segments scored as 2; more than nine segments scored as 3. The severity and extent of disease were then calculated as total HRCT score (range from 0 to 30). Each radiologist reviewed the scans independently of the other and a consensus opinion between them was taken in the event of disagreement. At the time of CT interpretation, the two radiologists (L.C. and M.C.) were not aware of the patient's history and physiologic results. The intraclass correlation coefficients for level of agreement between the radiologists on the total HRCT scores was 0.80 [18].

### Computer-aided scoring quantification process

**Fig 1:**
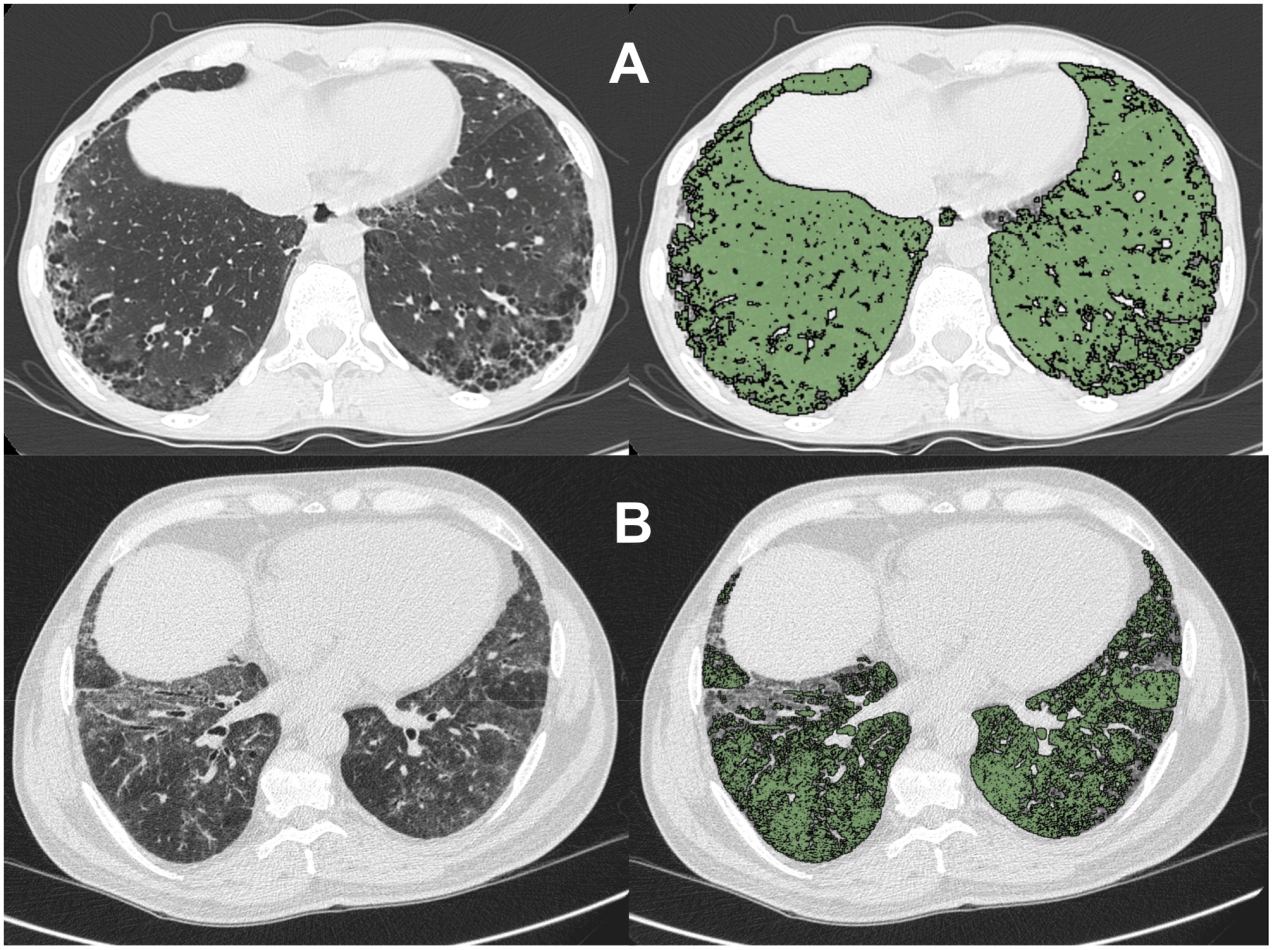
Representative sequences of the OsiriX segmentation process of two study participants. (A) Patient with mild (13%) of pulmonary fibrosis. (B) Patient with severe (41%) pulmonary fibrosis. We have developed the pulmonary fibrosis fraction by the following formula: total HRCT lung volume (−1.024 to −200)–nonfibrotic HRCT lung volume (−1.024 to –700) divided by total HRCT lung volume (−1.024 to −200) multiplied by 100.

HRCT images were reconstructed and analysed by OsiriX MD 7, a DICOM viewer software (OsiriX MD version 7, 64-bit format) on a Mac Mini (2.8 GHz Intel Core 2 Duo Desktop Computer, 16 GB random-access memory; Apple Computer, Cupertino, CA, USA) running Mac Operating System OSX 10.12.2. The DICOM data were stored in the OsiriX 7 using the ‘‘Copy linked files to Database folder” under ‘‘file” in the OsiriX 7 dropdown menu. For each section, a semiautomatic lung parenchymal segmentation was performed in order to obtain analysis of all images; then, descriptive parameters of the computer analysis, was calculated. This program uses a semiautomated thresholding technique to isolate the lungs from other tissues and structures and selects all pixels between -200 and -1.024 HU [14, 16]. Minimal user intervention was required to exclude blood vessels and large bronchi near the hilum. CT attenuation of normal lung parenchyma is reported to range from -800 to -900 HU, depending on ispiration or expiration, on the level of inspiration achieved for the scan and on anatomical location, that is ventral or dorsal portion [29–31]. The area with attenuation between -500 and -700 HU was defined as the value of radiodensity for ILD, including both ground-glass opacity and reticular opacity [16]. Moreover, the radiodensity of -500 HU was selected as the thresholds between consolidation and ground glass opacity [14]. Therefore, in agreement with Shin et al. [16] −700 HU is selected as the predefined threshold value for regions of normal lung. Fig 1 shows the representative sequences of the OsiriX segmentation process of two study participants with mild (A) and severe (B) pulmonary fibrosis, respectively. As mentioned before [18], there was total concordance between the first and second measurements on CaM scores (95% limits of agreement = 0 to 0, ICC = 1).

### Statistical analysis

All data was entered into a Microsoft Access database developed for the management of all data. The data was analysed using the MedCalc^®^ version 16.0 (MedCalc Software, Mariakerke, Belgium). Values in this study were expressed both as mean±SD (standard deviation) and median (interquartile range, IQR). A two-sample “t” test was used to compare continuous variables and the χ2 test was used to compare categorical variables between patients. The relationships among the lung segmentation analysis, the readers and the PFTs results were calculated using univariate regression analysis and Pearson’s product moment correlation (Pearson r values). Furthermore, multivariate regression analyses were performed to identify fctors associated with higher percentage of pulmonary fibrosis on CaM and with CoVR scores. Covariates entered into the models included age, sex, disease duration, anti-topoisomerase I antibodies, mRSS, Borg score, FVC and DLco (predicted). The VAS for breathing was excluded due to collinearity with Borg score. The results were expressed as multivariate regression coefficient (R) and square regression coefficient corrected (R^2^) for the number of variables entered in the analysis. This enables the calculation and the predictivity of each multivariate model according to the number of variables entered in the model itself. Significance was set at p <0.05.

## Results

The baseline characteristics of the 126 SSc patients are summarized in Table 1. Patients ranged in age from 22 years to 78 years, 83.7% were women. The mean±SD duration of the disease was 11.15±7.96 years. Anti-centromere antibodies were present in 55 patients (43.6%) and anti-topoisomerase I antibodies in 41 (32.5%).

**Table 1:** Baseline characteristics of the patients.

	Mean	SD	Median	IQR
Demographic data:				
• Age, years	60.68	10.74	61.00	54.00–68.00
• Disease duration, years	11.15	7.96	10.00	5.00–15.00
Clinical variables:				
• mRSS	10.22	7.37	7.00	5.00–14.00
• Borg score	2.59	1.62	2.00	1.00–4.00
• VAS for breathing	29.24	17.64	30.00	20.00–40.00
• HAQ-DI	0.84	0.35	0.87	0.62–0.92
Autoantibodies pattern:				
• Anti-topoisomerase I, n (%)	41 (32.5)			
• Anti-centromere, n (%)	55 (43.6)			
Pulmonary function tests:				
• DLco, % predicted	71.59	14.39	72.80	59.00–84.50
• FVC, % predicted	80.69	18.63	83.00	64.10–94.00
HRCT—Conventional Visual Reader-Based Score (CoVR) Quantification:				
• HRCT extent	6.30	3.46	5.00	3.00–9.00
• HRCT severity	6.96	3.71	6.00	4.00–10.00
• HRCT total score	13.22	6.98	11.00	8.00–19.00
HRCT—Computer-Aided Method (CaM) Quantification:				
• Pulmonary fibrosis (%)	12.68	7.99	10.05	6.39–17.84

Abbreviations: mRSS = modified Rodnan Skin Score; VAS = Visual Analog Scale; HAQ-DI = Health Assessment Questionnaire-Disability Index; DLCO = Single Breath Carbon Monoxide Diffusing Capacity of the Lung; FVC = Forced Vital Capacity; HRCT = High Resolution Computed Tomography.

On average, the HAQ-DI indicated only mild functional abnormalities (mean±SD 0.84±0.35). On PFTs, average FVC was 80.69±18.63% of predicted and average DLco was 71.59±14.39% of predicted. On HRCT, 116 patients (94.3%) displayed findings of ILD (detected by the readers: average total HRCT score = 12.1±6.9). The mean (±SD) time interval between PFTs and HRCT was 5.2±1.9 days (range: 0–7 days). Patients with dcSSc had a higher HAQ-DI, compared to those with limited cutaneous disease (1.00±0.33 vs. 0.71±0.32; p <0.05). Fifty-five patients were classified as having dcSSc and 71 patients were classified as having lcSSc (mean age, 59.67±11.04 years; range, 31 to 69 years; disease duration, 10.70±8.56 years). The group of patients having dcSSc, in comparison with lcSSc patients, was older (61.98±10.30 vs. 59.67±11.04 years; p <0.05) and with a long-term disease (11.74±7.15 vs. 10.70±8.56 years; p <0.05). The percentage of anti-topoisomerase I antibodies was significantly higher (41.8% vs. 25.4%; p = 0.03), in patients with dcSSc, whereas the percentage of anti-centromere antibodies was not significantly higher in patients with lcSSc (47.8% vs. 38.2%; p = 0.07). FVC and DLco were not statistically different in the two groups of SSc patients (p <0.001). Similarly, the percentage of pulmonary fibrosis measured by CaM was not significantly higher, in patients with dcSSc (14.01±8.43% vs. 11.64±7.54; p = 0.008) (Table 2).

**Table 2:** Comparison among baseline characteristics in SSc patients with diffuse cutaneous disease (n = 55) and limited cutaneous disease (n = 71).

	SSc type							
	Diffuse SSc				Limited SSc			
	Mean	SD	Median	IQR	Mean	SD	Median	IQR
Demographic data:								
• Age, years	61.98	10.30	65.00	54.25–69.00	59.67	11.04	59.00	54.00–67.75
• Disease duration, years	11.74	7.15	10.00	7.00–15.75	10.70	8.56	9.00	5.00–13.00
Clinical variables:								
• mRSS	16.87	6.08	15.00	12.25–21.00	4.85	2.14	5.00	4.00–6.00
• Borg score	2.81	1.60	3.00	1.25–4.00	2.42	1.63	2.00	1.00–3.00
• VAS for breathing	32.09	19.57	30.00	20.00–40.00	28.02	15.91	30.00	20.00–30.00
• HAQ-DI	1.00	0.33	0.92	0.87–1.12	0.710	0.32	0.62	0.500–0.87
Autoantibodies pattern:								
• Anti-topoisomerase I, n (%)	23 (41.8)				18 (25.4)			
• Anti-centromere, n (%)	21 (38.2)				34 (47.8)			
Pulmonary function tests:								
• DLco, % predicted	69.00	15.24	67.00	56.00–80.40	73.60	13.47	76.10	66.12–84.85
• FVC, % predicted	78.52	17.68	80.00	63.20–90.90	82.36	19.29	84.00	64.40–96.37
HRCT—Conventional Visual Reader-Based Score (CoVR) Quantification:								
• HRCT extent	6.89	3.39	7.00	4.00–10.00	5.84	3.47	5.00	3.00–8.00
• HRCT severity	7.63	3.81	7.00	4.25–10.00	6.43	3.57	5.00	4.00–7.75
• HRCT total score	14.45	7.01	14.00	8.00–20.00	12.26	6.86	10.00	7.00–14.75
HRCT—Computer-Aided Method (CaM) Quantification:								
• Pulmonary fibrosis (%)	14.01	8.43	11.03	7.12–18.86	11.64	7.54	8.09	5.99–17.07

For abbreviations see Table 1.

**Fig 2:**
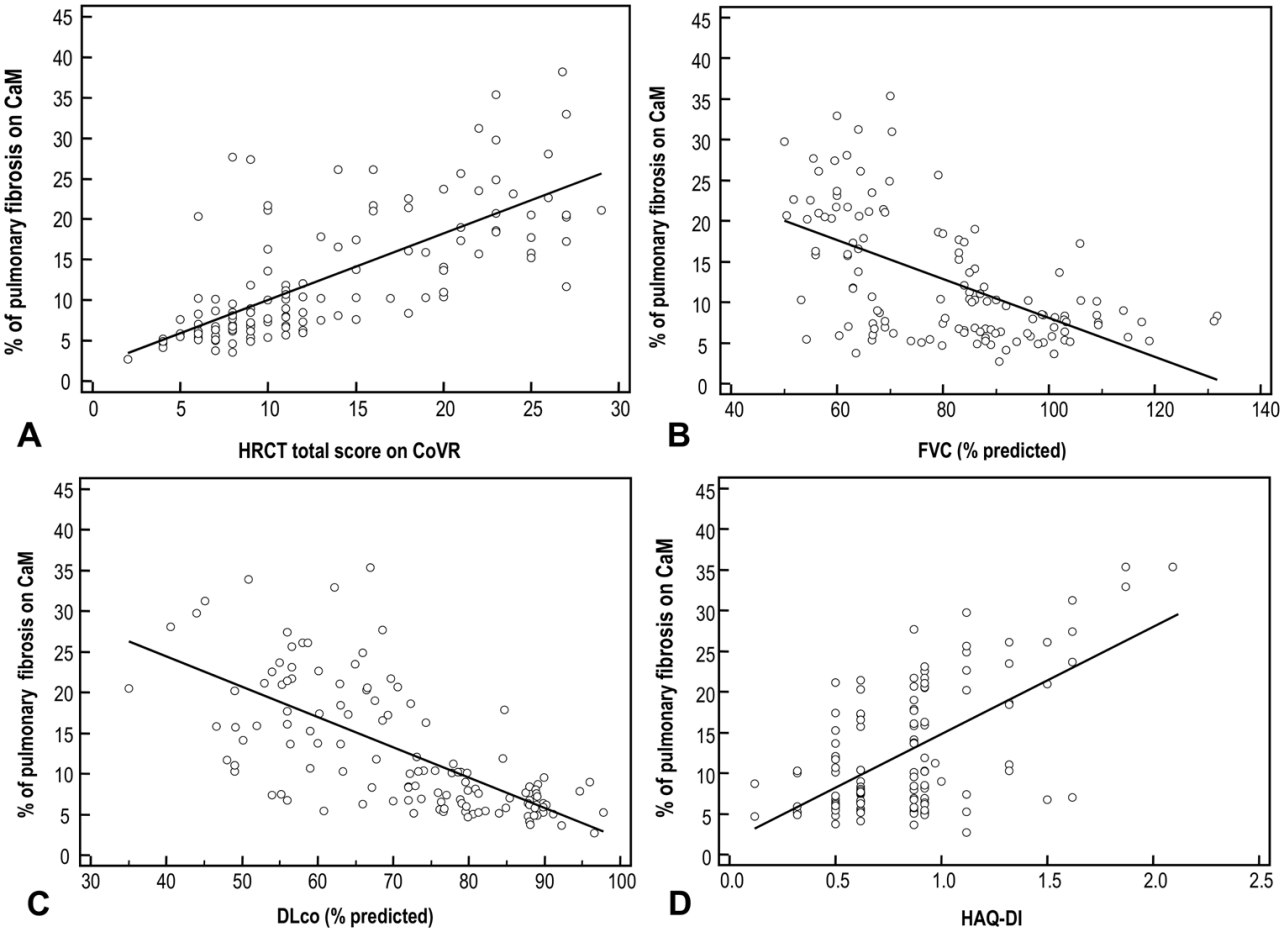
Scatter plots with regression line. (A) Correlation between computer-aided method (CaM) system for quantification of ILD on HRCT in SSc and the conventional visual reader-based score (CoVR). (B) Correlation between percentage of pulmonary fibrosis on CaM system and FVC, % predicted. (C) Correlation between percentage of pulmonary fibrosis on CaM system and DLco, % predicted. (D) Correlation between percentage of pulmonary fibrosis on CaM system and HAQ-DI.

A close correlation between CaM score results and CoVR total score was observed (Pearson r = 0.718; p <0.0001) (Fig 2A). The CaM scores showed a highly significant negative correlation with FVC (Pearson r -0.556; p <0.0001) (Fig 2B) and the DLco (Pearson r -670; p <0.0001) (Fig 2C). The Borg score and VAS for breathing were highly correlated with each other (Pearson r 0.627). Borg score and VAS for breathing were also significantly correlated (p <0.0001) with HAQ-DI (Pearson r 0.546 and 0.627, respectively). The HAQ-DI was further significantly correlated with CaM score (Pearson r 0.597; p <0.0001) (Fig 2D) and CoVR total score results (Pearson r 0.388; p <0.0001) and with mRSS (Pearson r 0.468; p <0.0001). The FVC and DLco were only weakly correlated with the HAQ-DI, and showed no correlations with the mRSS.

The results of the multivariate regression analysis indicate that the combination of DLco, FVC, Borg score and HAQ-DI explained the 83.4% of variance of percentage of pulmonary fibrosis on CaM-based method, whereas the variance of the CoVR total score was independently predicted by the same variables, with a lower coefficient of determination R^2^ (77.9% of variance) (Table 3). Age, sex, disease duration, anti-topoisomerase I antibodies and mRSS were not significantly associated with percentage of pulmonary fibrosis on CaM and with CoVR scores.

**Table 3:** Summary of the results of multiple regression analysis, with regression coefficients for the predictor variables on CaM results and CoVR scores.

Regression equation for CaM scores (dependent variable)					
Independent variables	Coefficient	Std. Error	r_partial_	t	P
Age, years	0.0244	0.0298	0.0769	0.820	0.4138
Sex	0.0415	0.0465	0.0836	0.892	0.3743
Disease duration, years	-0.0427	0.0220	-0.1103	-2.189	0.0663
Anti-topoisomerase I antibodies	0.0415	0.0464	0.0836	0.892	0.3743
mRSS	-0.0299	0.0278	-0.1007	-1.076	0.2844
HAQ-DI	2.3048	1.0412	0.2236	2.444	0.0117
Borg score	1.6900	0.4722	0.3191	3.579	0.0009
FVC, predicted	-0.0927	0.0420	-0.2033	-2.207	0.0193
DLco, predicted	-0.0453	0.0169	-0.2443	-2.678	0.0085
Regression equation for CoVR total scores (dependent variable)					
Independent variables	Coefficient	Std. Error	r_partial_	t	P
Age, years	0.0543	0.0476	0.1067	1.141	0.2562
Sex	0.2822	1.1444	0.0231	0.247	0.8057
Disease duration, years	-0.0405	0.0744	-0.0511	-0.545	0.5870
Anti-topoisomerase I antibodies	0.0543	0.0476	0.1067	1.141	0.2562
mRSS	-0.0780	0.0645	-0.1121	-1.210	0.2286
HAQ-DI	4.4489	1.5783	0.2542	2.819	0.0057
Borg score	1.2445	0.3345	0.2980	3.227	0.0019
FVC, predicted	-0.0508	0.0247	-0.1888	-2.043	0.0363
DLco, predicted	-0.1103	0.0321	-0.2439	-3.030	0.0043

For abbreviations see Table 1.

## Discussion

The pulmonary involvement is a serious complication of SSc [32, 33]. CT features of fibrosis are present in 55% to 65% of all patients with SSc and in up to 96% of those with abnormal PFT results [34–36]. As a result, HRCT has become an important part of the routine evaluation of ILD, and, in conjunction with PFT, plays a critical role in the treatment of ILD and in the prediction of outcomes [12, 37–40].

Semi-quantitative scoring methods, by grading each abnormality along with the bronchopulmonary segments involved [10–12], compared to quantitative scores using computer-based approaches have been investigated. To date, several computer tools to segment the lung automatically using HRCT images have been developed [15, 40, 41]. They include image display (e.g., multiplanar reformations and surface shading for three-dimensional and volume rendering), anatomic image quantification (e.g., area and volume of airways and lungs) and regional characterization of lung tissue (analyzing attenuation, changes in attenuation, and texture patterns in the imaged lung) [15, 42–44]. Computer-based models correlate well with visual scoring techniques for the detection of fibrosis and with the assessment of extent of disease, without the intrareader variation encountered with visual scoring [15–17]. Recently, we showed that the CaM may assist the rheumatologist analysis of lung HRCT data and it provides an objective method for supplementing subjective visual-based grading of the extent of ILD to achieve precise and independent reader quantification [18].

On HRCT, 116 patients (94.3%) displayed findings of ILD. Our results showed, in agreement with other authors [45], that the percentage of fibrosis measured by CaM, was not significantly higher in patients with dcSSc. Patiwetwitoon et al. compared the HRCT findings between patients with dcSSc and lcSSc and found that the HRCT scores of these patients were comparable in both subtypes of SSc [45]. In the Scleroderma Lung Study, lcSSc and dcSSc patients were indistinguishable with regard to their baseline pulmonary functions, but lcSSc patients presented more extensive pulmonary fibrosis. Furthermore the rate of progression of ILD is similar in lcSSc and dcSSc patients [46].

In univariate analysis, we observed a strong correlation between percentage of pulmonary fibrosis on CaM and CoVR scores (p <0.0001). The HRCT CaM and CoVR scores were statistically related to severity of functional lung parameters. The percentage of pulmonary fibrosis on CaM showed a more significant negative correlation with FVC and the DLco. In multivariate analysis, the CaM and CoVR scores were predicted by DLco, FVC, Borg score, and HAQ-DI. Age, sex, disease duration, anti-topoisomerase I antibodies and mRSS were not significantly associated with percentage of pulmonary fibrosis on CaM and CoVR scores. These observations suggest that parenchymal lung disease in SSc-ILD may have a high impact on gas transfer and is consistent with prior studies [7, 36, 47]. In this respect, Tashkin et al. [37], using baseline data from two large, randomised, interventional studies (Scleroderma Lung Study I and II, or SLS I and II), found that DLco was the single variable that best correlated with the extent of both lung fibrosis and total ILD (more so than the FVC), when assessed both in the zone of maximal involvement and in the whole lung. Similarly, DLco was better correlated with the radiographic extent of lung fibrosis and total ILD than FVC% in bivariate analyses.

Change in DLco is influenced equally by the integrity of the alveolar–capillary interface, as well as ventilation (including alveolar volume), and perfusion (including hemoglobin). Thus, in SSc, where reduced volumes generally indicate the presence of ILD, impaired DLco is not specific and can indicate the presence, in varying degrees of ILD, pulmonary hypertension, and other disease manifestations, including anemia. In addition, DLco has marked measurement variability both within each testing session and between sessions.

Functional disability is considerable in SSc [1, 3, 26, 27], and may be influenced by respiratory impairment and other factors, such as the extent of skin involvement, tendon/joint contracture, damage in the heart, and peripheral vascular system. The median HAQ-DI seen in our cohort was 0.84, which is in keeping with the reported range of this index in SSc patients of 0.83–1.2 [48, 49]. We were able to show that patients with lcSSc had significantly less disability than those with dcSSc. Moreover, in apparent contrast to recent findings [47], we found that the HAQ-DI, correlated with PFT, subjective patient-oriented measures of dyspnea and HRCT scores, suggested that the HAQ-DI should be included as surrogate outcome measure of HRCT-defined severity of fibrosis in this population. In this respect, Volkmann et al., using data from the SLS-I [50], found that a composite measure comprised of variables included the FVC, computer-based score for quantitative lung fibrosis in the zone of maximum fibrosis from HRCT scans, transitional dyspnea index, and the HAQ-DI may serve as a more comprehensive measure of cyclophosphamide treatment effect in SSc-ILD compared with a single outcome approach (e.g., FVC predicted).

The most important limitations are as follows. First, and probably the most important, this is a cross-sectional study; therefore, it is unknown whether the observed relationships persist over time. Second, we did not include a respiratory disease-specific questionnaire, such as the St. George’s Respiratory Questionnaire, which would have further strengthened our study [51]. Third, pulmonary hypertension could not be strictly excluded based on clinical findings or clinical history alone in patients with ILD-SSc. Another limitation is that using a data set from a single centre to select the regression models, may overestimate the model association performances when applied to other populations.

In conclusion, taken together, the results indicate that, although a close correlation between CaM score results and CoVR total score was found, CaM results showed a more significant correlation with DLco (more so than FVC), patient-centred measures of perceived dyspnea and functional disability in comparison to CoVR scores. A computer-based quantification system is computationally efficient and provides the best overall estimates of HRCT-measured lung disease. The visual-based scoring techniques offer undisputed advantage in finding type and extent of lung anatomical-functional damage caused by SSc, or in distinguishing other causes of increased lung density, such as infection or neoplasm. Visual and computer-based quantitative scoring system are complementary, rather than competitive [35]. In combination with physiologic parameters and patient-centred measures of perceived dyspnea and functional disability, a computer-based quantification system could be a means for accurately assessing and monitoring the disease progression or response to therapy [50]. In a future study, we will address the sensitivity of changes in whole lung fibrosis score over time, in the presence and absence of therapeutic intervention as a necessary validation step.

## Disclosures

This work was not supported by any research grants. All the authors have contributed substantially to this study, and there are no conflicts of interests to be declared.

## References

[1] Steen VD, Medsger TA Jr. Changes in causes of death in systemic sclerosis. 1972–2002. Ann Rheum Dis. 2007; 66(7): 940–944. 17329309 10.1136/ard.2006.066068 PMC1955114

[2] McNearney TA, Reveille JD, Fischbach M, Friedman AW, Lisse JR, Goel N, et al Pulmonary involvement in systemic sclerosis: associations with genetic, serologic, sociodemographic, and behavioural factors. Arthritis Rheum. 2007; 57(2): 318–326. 17330281 10.1002/art.22532

[3] Hudson M, Thombs BD, Steele R, Panopalis P, Newton E, Baron M, Canadian Scleroderma Research Group. Health-related quality of life in systemic sclerosis: a systematic review. Arthritis Rheum. 2009; 61(8): 1112–1120. 10.1002/art.24676 19644906

[4] Khanna D, Nagaraja V, Tseng CH, Abtin F, Suh R, Kim G, et al Predictors of lung function decline in scleroderma-related interstitial lung disease based on high-resolution computed tomography: implications for cohort enrichment in systemic sclerosis-associated interstitial lung disease trials. Arthritis Res Ther. 2015; 17(1):372 10.1186/s13075-015-0872-2 26704522 PMC4718035

[5] Goldin JG, Lynch DA, Strollo DC, Suh RD, Schraufnagel DE, Clements PJ, et al High resolution CT findings in scleroderma-related lung diseases: findings from Scleroderma Lung Study. Chest. 2008; 134(2): 358–367. 18641099 10.1378/chest.07-2444

[6] Remy-Jardin M, Remy J, Wallaert B, Bataille D, Hatron PY. Pulmonary involvement in progressive systemic sclerosis: sequential evaluation with CT, pulmonary function tests, and bronchoalveolar lavage. Radiology. 1993; 188(2): 499–506. 8327704 10.1148/radiology.188.2.8327704

[7] Diot E, Boissinot E, Asquier E, Guilmot JL, Lemarié E, Valat C, et al Relationship between abnormalities on high-resolution CT and pulmonary function in systemic sclerosis. Chest. 1998; 114(6): 1623–1629 9872198 10.1378/chest.114.6.1623

[8] Schurawitzki H, Stiglbauer R, Graninger W, Herold C, Pölzleitner D, Burghuber OC, et al Interstitial lung disease in progressive systemic sclerosis: high-resolution CT versus radiography. Radiology. 1990; 176(3):755–759. 2389033 10.1148/radiology.176.3.2389033

[9] Steen VD, Conte C, Owens GR, Medsger TA Jr. Severe restrictive lung disease in systemic sclerosis. Arthritis Rheum. 1994; 37(9): 1283–1289. 7945490 10.1002/art.1780370903

[10] Kazerooni EA, Martinez FJ, Flint A, Jamadar DA, Gross BH, Spizarny DL, et al Thin-section CT obtained at 10-mm increments versus limited three-level thin-section CT for idiopathic pulmonary fibrosis: correlation with pathologic scoring. AJR Am J Roentgenol. 1997; 169(4): 977–983. 9308447 10.2214/ajr.169.4.9308447

[11] Warrick JH, Bhalla M, Schabel SI, Silver RM. High resolution computed tomography in early scleroderma lung disease. J Rheumatol. 1991; 18(10): 1520–1528. 1765976

[12] Goh NS, Desai SR, Veeraraghavan S, Hansell DM, Hoyles RK, Sato H, et al Interstitial lung disease in systemic sclerosis: a simple staging system. Am J Respir Crit Care Med. 2008; 177(11): 1248–1254. 10.1164/rccm.200706-877OC 18369202

[13] Ooi GC, Mok MY, Tsang KW, Wong Y, Khong PL, Fung PC, et al Interstitial lung disease in systemic sclerosis. Acta Radiol. 2003; 44(3): 258–264. 12751995 10.1080/j.1600-0455.2003.00058.x

[14] Yabuuchi H, Matsuo Y, Tsukamoto H, Horiuchi T, Sunami S, Kamitani T, et al Evaluation of the extent of ground-glass opacity on high-resolution CT in patients with interstitial pneumonia associated with systemic sclerosis: comparison between quantitative and qualitative analysis. Clin Radiol. 2014; 69(7): 758–764. 10.1016/j.crad.2014.03.008 24824977

[15] Kim HG, Tashkin DP, Clements PJ, Li G, Brown MS, Elashoff R, et al A computer-aided diagnosis system for quantitative scoring of extent of lung fibrosis in scleroderma patients. Clin Exp Rheumatol. 2010; 28(5 Suppl 62): 26–35. PMC3177564 21050542

[16] Shin KE, Chung MJ, Jung MP, Choe BK, Lee KS. Quantitative computed tomographic indexes in diffuse interstitial lung disease: correlation with physiologic tests and computed tomography visual scores. J Comput Assist Tomogr. 2011; 35(2): 266–271. 10.1097/RCT.0b013e31820ccf18 21412102

[17] Ariani A, Carotti M, Gutierrez M, Bichisecchi E, Grassi W, Giuseppetti GM, et al Utility of an open-source DICOM viewer software (OsiriX) to assess pulmonary fibrosis in systemic sclerosis: preliminary results. Rheumatology International. 2014; 34(4): 511–516. 10.1007/s00296-013-2845-6 23949623

[18] Salaffi F, Carotti M, Bosello S, Ciapetti A, Gutierrez M, Bichisecchi E, et al Computer-aided quantification of interstitial lung disease from high resolution computed tomography images in systemic sclerosis: correlation with visual reader-based score and physiologic tests. BioMed Research International. 2015; 10.1155/2015/834262 PMC4299560 25629053

[19] Subcommittee for Scleroderma Criteria of the American Rheumatism Association Diagnostic and Therapeutic Criteria Committee. Preliminary criteria for the classification of systemic sclerosis (scleroderma). Arthritis Rheum. 1980; 23(5): 581–590. 7378088 10.1002/art.1780230510

[20] LeRoy EC, Black C, Fleischmajer R, Jablonska S, Krieg T, Medsger TA Jr, et al Scleroderma (systemic sclerosis): classification, subsets and pathogenesis. J Rheumatol. 1988; 15(2): 202–205. 3361530

[21] Clements P, Lachenbruch P, Siebold J, White B, Weiner S, Martin R, et al Inter and intraobserver variability of total skin thickness score (modified Rodnan TSS) in systemic sclerosis. J Rheumatol. 1995; 22(7): 1281–1285. 7562759

[22] Borg GA. Psychophysical bases of perceived exertion. Med Sci Sports Exerc. 1982; 14(5): 377–381. 7154893

[23] Fries JF, Spitz P, Kraines RG, Holman HR. Measurement of patient outcome in arthritis. Arthritis Rheum. 1980; 23(2): 137–145. 7362664 10.1002/art.1780230202

[24] Poole JL, Steen VD. The use of the Health Assessment Questionnaire (HAQ) to determine physical disability in systemic sclerosis. Arthritis Care Res. 1991; 4(1): 27–31. 11188583 10.1002/art.1790040106

[25] Khanna D, Clements PJ, Furst DE, Chon Y, Elashoff R, Roth MD, et al Correlation of the degree of dyspnea with health-related quality of life, functional abilities, and diffusing capacity for carbon monoxide in patients with systemic sclerosis and active alveolitis: results from the Scleroderma Lung Study. Arthritis Rheum. 2005; 52(2): 592–600. 15692967 10.1002/art.20787

[26] Clements PJ, Wong WK, Hurwitz EL, Furst DE, Mayes M, White B, et al Correlates of the disability index of the Health Assessment Questionnaire: a measure of functional impairment in systemic sclerosis. Arthritis Rheum. 1999; 42(11): 2372–2380. 10555033 10.1002/1529-0131(199911)42:11<2372::AID-ANR16>3.0.CO;2-J

[27] Clements PJ, Wong WK, Hurwitz EL, Furst DE, Mayes M, White B, et al The disability index of the Health Assessment Questionnaire is a predictor and correlate of outcome in the high-dose versus low-dose penicillamine in systemic sclerosis trial. Arthritis Rheum. 2001; 44(3): 653–661. 11263780 10.1002/1529-0131(200103)44:3<653::AID-ANR114>3.0.CO;2-Q

[28] Miller MR, Hankinson J, Brusasco V, Burgos F, Casaburi R, Coates A, et al Standardisation of spirometry. Eur Respir J. 2005; 26(2): 319–338. 16055882 10.1183/09031936.05.00034805

[29] Wegener OH, Koeppe P, Oeser PH. Measurement of lung density by computed tomography. J Computed Assist Tomograf. 1978; 2(3): 263–273. 10.1097/00004728-197807000-00003 263489

[30] Robinson PJ, Kreel L. Pulmonary tissue attenuation with computed tomography: comparison of inspiration and expiration scans. J Computed Assist Tomograf. 1979; 3(6): 740–748. 512106

[31] Rosemblum LJ, Mauceri RA, Wellenstein DE, Thomas FD, Bassano DA, Raasch BN, et al Density pattern in the normal lung as determined by computed tomography. Radiology. 1980; 137(2): 409–416. 7433674 10.1148/radiology.137.2.7433674

[32] Mathai SC, Hummers LK, Champion HC, Wigley FM, Zaiman A, Hassoun PM, et al Survival in pulmonary hypertension associated with the scleroderma spectrum of diseases: impact of interstitial lung disease. Arthritis Rheum. 2009; 60(2): 569–577. 10.1002/art.24267 19180517

[33] Solomon JJ, Olson AL, Fischer A, Bull T, Brown KK, Raghu G. Scleroderma lung disease. Eur Respir Rev. 2013; 22(127): 6–19. 10.1183/09059180.00005512 23457159 PMC4103193

[34] Fujita J, Yoshinouchi T, Ohtsuki Y, Tokuda M, Yang Y, Yamadori I, et al Non-specific interstitial pneumonia as pulmonary involvement of systemic sclerosis. Ann Rheum Dis. 2001; 60(3): 281–283. 11171693 10.1136/ard.60.3.281 PMC1753571

[35] Launay D, Remy-Jardin M, Michon-Pasturel U, Mastora I, Hachulla E, Lambert M, et al High resolution computed tomography in fibrosing alveolitis associated with systemic sclerosis. J Rheumatol. 2006; 33(9): 1789–1801. 16960939

[36] Wells AU, Rubens MB, Bois RM, Hansell DM. Serial CT in fibrosing alveolitis: prognostic significance of the initial pattern. AJR Am J Roentgenol. 1993; 161(6): 1159–1165. 8249719 10.2214/ajr.161.6.8249719

[37] Tashkin DP, Elashoff R, Clements PJ, Goldin J, Roth MD, Furst DE, et al Cyclophosphamide versus placebo in scleroderma lung disease. N Engl J Med. 2006; 354(25): 2655–2666. 16790698 10.1056/NEJMoa055120

[38] Roth MD, Tseng CH, Clements PJ, Furst DE, Tashkin DP, Goldin JG, et al Scleroderma Lung Study Research Group. Predicting treatment outcomes and responder subsets in scleroderma-related interstitial lung disease. Arthritis Rheum. 2011; 63(9): 2797–2808. 10.1002/art.30438 21547897 PMC3910296

[39] Khanna D, Brown KK, Clements PJ, Elashoff R, Furst DE, Goldin J, et al Systemic sclerosis-associated interstitial lung disease-proposed recommendations for future randomized clinical trials. Clin Exp Rheumatol. 2010; 28(2 Suppl 58): 55–62. 20576216

[40] Camiciottoli G, Orlandi I, Bartolucci M, Meoni E, Nacci F, Diciotti S, et al Lung CT densitometry in systemic sclerosis, correlation with lung function, exercise test and quality of life. Chest. 2007; 131(3): 672–681. 17356079 10.1378/chest.06-1401

[41] Pu J, Roos J, Yi CA, Napel S, Rubin GD, Paik DS. Adaptive border marching algorithm: automatic lung segmentation on chest CT images. Comput Med Imaging Graph. 2008; 32(6): 452–462. 10.1016/j.compmedimag.2008.04.005 18515044 PMC2536655

[42] Wang J, Li F, Li Q. Automated segmentation of lungs with severe interstitial lung disease in CT. Med Phys. 2009; 36(10): 4592–4599. 19928090 10.1118/1.3222872 PMC2771715

[43] Sverzellati N, Zompatori M, De Luca G, Chetta A, Bnà C, Ormitti F, et al Evaluation of quantitative CT indexes in idiopathic interstitial pneumonitis using a low-dose technique. Eur J Radiol. 2005; 56(3): 370–375. 15978764 10.1016/j.ejrad.2005.05.012

[44] Hartley PG, Galvin JR, Hunninghake GW, Merchant JA, Yagla SJ, Speakmann SB, et al High resolution CT-derived measures of lung density are valid indexes of interstitial lung disease. J Appl Physiol. 1994; 76(1): 271–277. 8175517 10.1152/jappl.1994.76.1.271

[45] Patiwetwitoon S, Wangkaew S, Euathrongchit J, Kasitanon N, Louthrenoo W. High-resolution computed tomographic findings in systemic sclerosis-associated interstitial lung disease: comparison between diffuse and limited systemic sclerosis. J Clin Rheumatol. 2012; 18(5): 229–233. 10.1097/RHU.0b013e318261176f 22832288

[46] Clements PJ, Roth MD, Elashoff R, Tashkin DP, Goldin J, Silver RM, et al Scleroderma Lung Study (SLS): differences in the presentation and course of patients with limited versus diffuse systemic sclerosis. Ann Rheum Dis. 2007; 66(12): 1641–1647. 17485423 10.1136/ard.2007.069518 PMC2095310

[47] Tashkin DP, Volkmann ER, Tseng CH, Kim HJ, Goldin J, Clements P, et al Relationship between quantitative radiographic assessments of interstitial lung disease and physiological and clinical features of systemic sclerosis. Ann Rheum Dis. 2014 12 1 pii: annrheumdis-2014-206076. 10.1136/annrheumdis-2014-206076 25452309

[48] Poole JL, Williams CA, Bloch DA, Hollak B, Spitz P. Concurrent validity of the Health Assessment Questionnaire Disability Index in Scleroderma. Arthritis Care Res. 1995; 8(3): 189–193. 7654804 10.1002/art.1790080312

[49] Strickland G, Pauling J, Cavill C, McHugh N. Predictors of health-related quality of life and fatigue in systemic sclerosis: evaluation of the EuroQol-5D and FACIT-F assessment tools. Clin Rheumatol. 2012; 31(8): 1215–1222. 10.1007/s10067-012-1997-1 22588647

[50] Volkmann ER, Tashkin DP, Li N, Furst DE, Clements P, Elashoff R. Development of a Composite Outcome Measure for Systemic Sclerosis Related Interstitial Lung Disease. Rheumatology (Sunnyvale). 2015; 5:154, 10.4172/2161-1149.1000154 28856069 PMC5573239

[51] Beretta L, Santaniello A, Lemos A, Masciocchi M, Scorza R. Validity of the Saint George's Respiratory Questionnaire in the evaluation of the health-related quality of life in patients with interstitial lung disease secondary to systemic sclerosis. Rheumatology (Oxford). 2007; 46(2): 296–301. 16877463 10.1093/rheumatology/kel221

